# Rapidly growing nodule on the knee

**DOI:** 10.1016/j.jdcr.2023.11.021

**Published:** 2023-12-04

**Authors:** Kirsten M. Johnson, Ania Henning, Jose A. Plaza, Alisha N. Plotner

**Affiliations:** a Department of Dermatology, The Ohio State University, Columbus, Ohio; b Department of Pathology, The Ohio State University, Columbus, Ohio

**Keywords:** fibromyxoid stroma, ganglion-like cells, myofibroblasts, proliferative fasciitis, pseudosarcomatous, subcutaneous nodule

A 51-year-old woman presents with spontaneous development of a nontender “bump” on her right knee that has been growing for approximately 1 year. Previous ultrasound did not lead to a diagnosis. Examination revealed a smooth, mobile, skin-colored, 1-cm subcutaneous nodule on her right knee (Fig 1). Excisional biopsy was performed, and histopathology revealed an ill-defined cellular proliferation of spindle fibroblasts and myofibroblasts arranged in short fascicles set within a myxoid-to-collagenous stroma. Numerous basophilic ganglion-like cells were noted in addition to a patchy lymphoplasmacytic inflammatory response. These ganglion-like cells had abundant eosinophilic cytoplasm with eccentrically located nuclei and prominent nucleoli (Figs 2 and 3). Mitoses were not increased. Immunohistochemically, the spindle cells were positive for SMA and CD163 but negative for S100, GFAP, AE1/AE3, desmin, and CD34.

**Fig 1:**
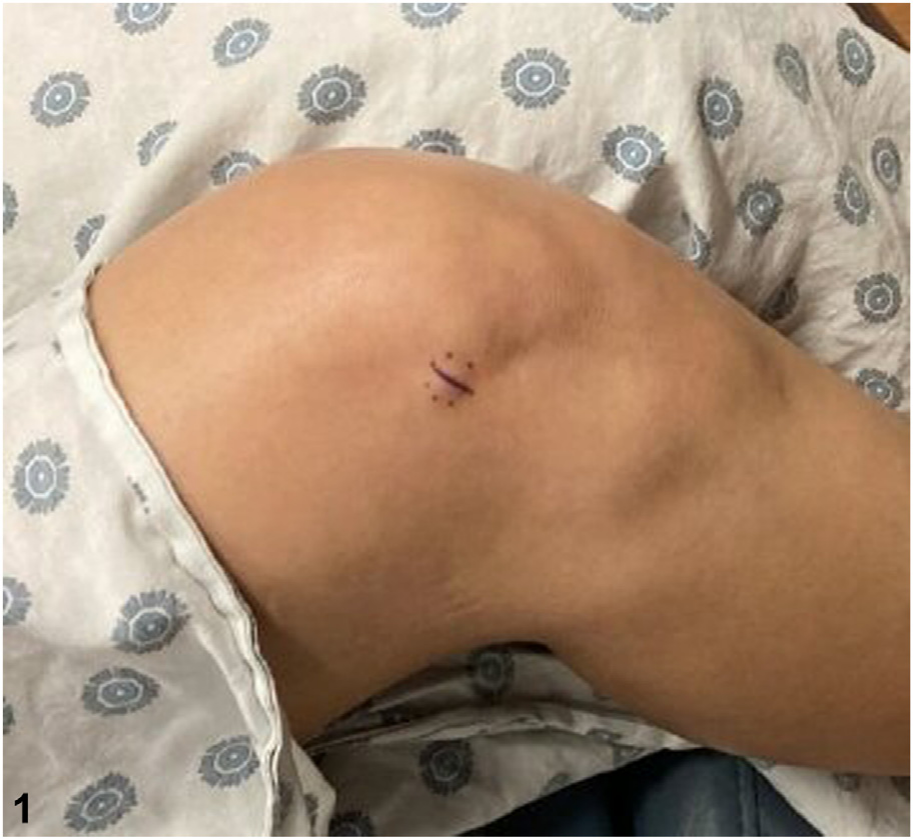

**Fig 2:**
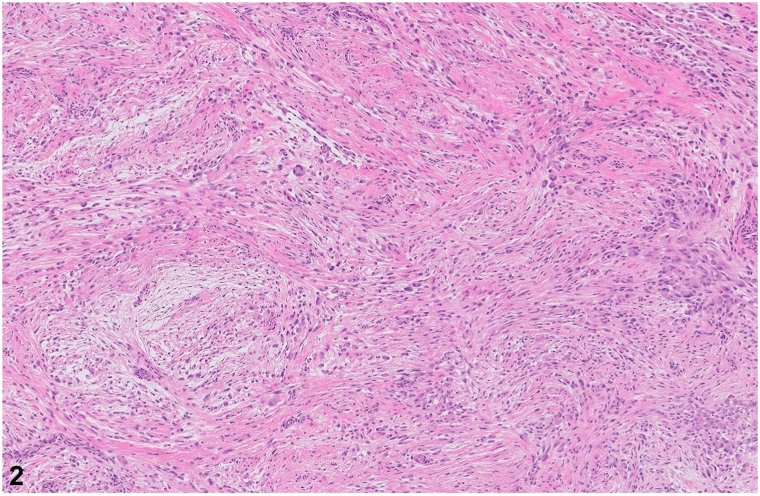

**Fig 3:**
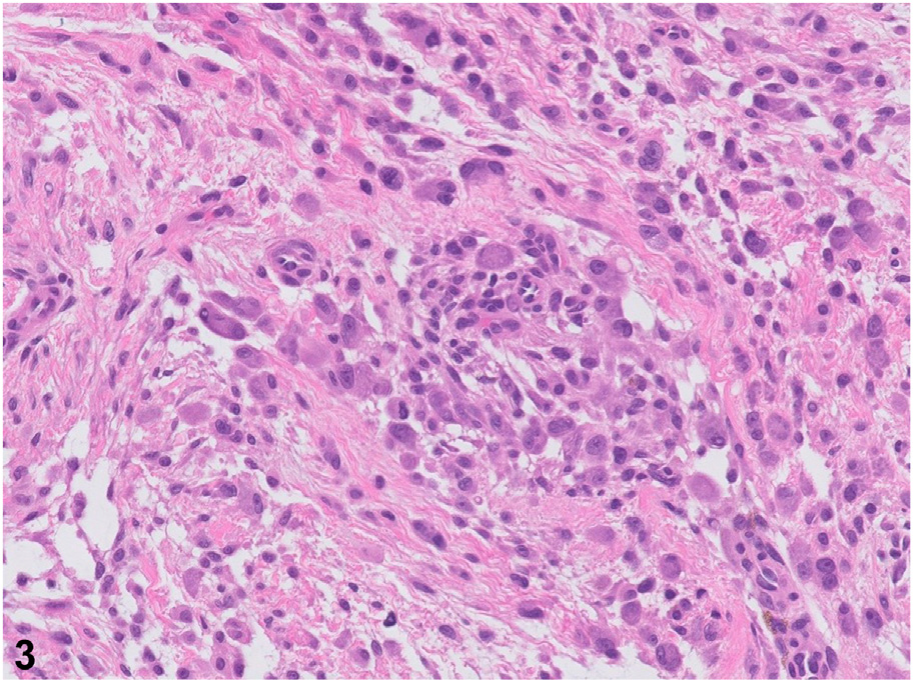

**Question 1: What is the most likely diagnosis?**

A. Atypical fibrous histiocytoma
B. Proliferative fasciitis
C. Proliferative myositis
D. Nodular fasciitis
E. Fibrosarcoma

**Answers:**

A. Atypical fibrous histiocytoma – Incorrect. This is a rare variant of dermatofibroma, which can be difficult to distinguish histologically from malignant tumors because of the striking atypia and nuclear pleomorphism of the atypical fibrous histiocytic cells. Clinically, it presents as a slow-growing brown-to-purple firm papule or nodule, in contrast to the subcutaneous nodule seen in this case.
B. Proliferative fasciitis – Correct. A rapidly growing subcutaneous nodule on the lower extremity with histological demonstration of myofibroblastic or fibroblastic proliferation and basophilic ganglion-like cells is most consistent with proliferative fasciitis.^1^ This pseudosarcomatous lesion is considered benign, although it demonstrates clinical and histological features similar to those of soft tissue sarcomas. Histopathology and immunohistochemical staining can help rule out spindle cell malignant neoplasms in the differential diagnosis. Microscopically, the diffuse proliferation of spindled fibroblastic cells and large basophilic ganglion-like cells is very characteristic of proliferative fasciitis (Fig 3).^2^
C. Proliferative myositis – Incorrect. Although proliferative myositis is also a pseudosarcomatous lesion with many histologically similar features, it grows within muscle tissue or between muscle fibers, whereas proliferative fasciitis grows within the subcutaneous tissue or even laterally along the fascial planes.^2^
D. Nodular fasciitis – Incorrect. Nodular fasciitis, a benign subcutaneous nodule, is usually rapidly growing and has a predilection for the upper portion of the body. There are overlapping histopathologic features with proliferative fasciitis; however, it can be distinguished by areas of abundant myxoid stroma with a mixed inflammatory infiltrate and lack of ganglion-like cells.^3^
E. Fibrosarcoma – Incorrect. Fibrosarcoma is a rare malignant fibroblastic or mesenchymal tumor that presents as a painless, poorly defined mass in soft tissues, often on the lower extremities.^3^ It usually arises within the fascia, muscle, aponeuroses, or tendons. Histopathologic findings demonstrate the proliferation of atypical spindle cells arranged as intersecting fascicles in a “herringbone” pattern. Depending on the site of origin, the tumor cells may be positive for vimentin and muscle-specific actin.^2^

**Question 2: What is the most appropriate next step in management?**

A. Sentinel lymph node dissection for staging
B. Intralesional corticosteroids
C. Local radiation
D. Clinical observation
E. Re-excision with 5-mm margins

**Answers:**

A. Sentinel lymph node dissection for staging – Incorrect. As a benign neoplasm, there is no role for sentinel lymph node dissection or any staging for proliferative fasciitis.
B. Intralesional corticosteroids – Incorrect. This pseudosarcomatous lesion would show very little, if any, response to intralesional corticosteroids.
C. Local radiation – Incorrect. The treatment for fibrosarcoma depends on clinical staging; however, it often includes adjuvant radiation for larger or high-grade tumors.^3^ Anthracyclines are often used as chemotherapy of choice. Because proliferative fasciitis is a benign neoplasm, radiation is not indicated.
D. Clinical observation – Correct. Although proliferative fasciitis is rapidly growing and has some histological features that can be initially confused with soft tissue sarcomas because of their cellularity and immature appearance of proliferating cells, this is a benign neoplasm. Although clinical observation is the correct management for this rare entity, it is often excised for biopsy during the diagnostic work-up, as with our patient, to rule out the malignant conditions on the clinical differential diagnosis.^4^
E. Re-excision with 5-mm margins – Incorrect. As a benign neoplasm, clear margins are not required for proliferative fasciitis. Elective excision may be requested by the patient owing to appearance, rapid enlargement of the nodule, or symptoms such as pain, but it is not required.

**Question 3: What immunostain would differentiate proliferative fasciitis from atypical fibrous histiocytoma?**

A. S100
B. P63
C. SMA
D. Desmin
E. S100A6

**Answers:**

A. S100 – Incorrect. S100 is a marker for melanocytic differentiation and cells of neural crest origin.
B. P63 – Incorrect. P63 is a marker for epithelial tumors of the skin, including actinic keratoses, basal cell carcinoma, squamous cell carcinoma, and spindle cell squamous cell carcinoma. Staining for P63 in proliferative fasciitis would be negative.
C. SMA – Correct. Histopathology demonstrates spindle cells that were positive for smooth muscle marker (SMA) but negative for S100 (marker for melanocytic differentiation and neural crest origin), GFAP (maker that stains glial protein), AE1 or AE3 (cytokeratin markers for carcinomas), and desmin (myogenic stain).^2^^,^^5^ Atypical fibrous histiocytoma is a fibrohistiocytic neoplasm that would be positive for Factor XIIa and histiocytic markers (such as CD68) and negative for SMA.
D. Desmin – Incorrect. Desmin, a marker for smooth muscle, would stain negative in proliferative fasciitis.
E. S100A6 – Incorrect. This calcium-binding protein is highly expressed in epithelial cells, fibroblasts, and several different types of cutaneous and internal malignancies. It would not be useful for highlighting the spindle cell component of proliferative fasciitis.

## Conflicts of interest

The authors declare no conflicts of interest. Authors declare that the contents of this article are their own original unpublished findings.
